# Sarcopenia and sarcopenic obesity as prognostic predictors in hospitalized elderly patients with acute myocardial infarction

**DOI:** 10.31744/einstein_journal/2019AO4632

**Published:** 2019-08-14

**Authors:** Natália de Moraes Santana, Roberta Maria Lins Mendes, Nadja Fernandes da Silva, Cláudia Porto Sabino Pinho

**Affiliations:** 1 Universidade de Pernambuco, Recife, PE, Brazil

**Keywords:** Sarcopenia, Cardiovascular diseases, Myocardial infarction, Obesity, Aged

## Abstract

**Objective:**

To investigate the potential value of sarcopenia and sarcopenic obesity as prognostic predictors in hospitalized elderly patients with acute myocardial infarction.

**Methods:**

A cross-sectional study based on data collected from elderly patients with acute myocardial infarction, admitted to a public hospital located in the Northeastern region of Brazil, from April to July 2015. The diagnosis of sarcopenia was based on muscle mass, muscle strength and physical performance measurements. Cardiovascular risk and prognostic markers, such as troponin and creatine kynase MB isoenzyme values, acute myocardial infarction classification according to ST segment elevation, and thrombolysis in myocardial infarction score were used.

**Results:**

The sample comprised 99 patients with mean age of 71.6 (±7.4) years. Prevalence of sarcopenia and sarcopenic obesity was 64.6% and 35.4%, respectively. Sarcopenia was more prevalent among males (p=0.017) aged >80 years (p=0.008). Thrombolysis in myocardial infarction was the only marker of cardiovascular risk significantly associated with sarcopenia (p=0.002).

**Conclusion:**

Prevalence of sarcopenia was high and associated with thrombolysis in myocardial infarction risk score. Sarcopenic obesity affected approximately one-third of patients and was not associated with any of the prognostic predictors.

## INTRODUCTION

Sarcopenia is a multifactorial geriatric syndrome, defined as progressive loss of muscle mass associated with reduced muscle strength and/or physical performance. Affected individuals may experience motor function compromise and loss of autonomy, with reduced quality of life and increased morbidity and mortality risks.^(^^1^^–^^3^^)^ Data on the incidence and prevalence of sarcopenia are scarce in Brazilian scientific literature. The prevalence of sarcopenia ranges from 5 to 50%;^(^^3^^)^ differences in reported prevalence may be due to a variety of factors, including different ethnic composition of samples, adoption of different criteria to define sarcopenia and/or different lean mass measurement methods.

Metabolic and cardiovascular risks (CVR) are closely related to aging and there is a growing interest in the study of associated factors, given cardiovascular diseases (CVD) are a major global cause of disability and death. Consistent associations between low muscle mass and risk of mortality have been demonstrated in some studies; however, potential associations between sarcopenia and CVD risk remain to be confirmed.^(^^4^^,^^5^^)^

A recent review of clinical observational studies revealed associations between low muscle mass and arterial stiffness, an independent predictor of CVD. Also, sarcopenia may promote atherogenesis due to relative fat mass increase in response to loss of muscle mass and replacement of myocytes by adipocytes.^(^^6^^)^ Hence, sarcopenia in the presence of excess fat tissue, or sarcopenic obesity (SO), would have an even greater impact on metabolic diseases, CVD and mortality compared to obesity or sarcopenia alone.^(^^7^^)^

Global aging population growth emphasizes the need to focus on adverse age-related conditions, such as sarcopenia and SO, as these are directly associated with loss of autonomy and potentially poorer cardiovascular prognosis.

## OBJECTIVE

To investigate associations between sarcopenia and sarcopenic obesity as prognostic predictors in elderly patients with acute myocardial infarction.

## METHODS

Retrospective study carried out at a public hospital, which is a reference in cardiology, located in the Northeastern region of Brazil, based on data collected from April to June 2015, and later, within two years of the first hospitalization. The sample comprised male and female patients aged ≥60 years, and hospitalized in the coronary care unit. Patients with physical (amputees) or cognitive (no eye contact with interviewer) impairments, bedridden, recovering from cardiac surgery, suffering from kidney disease and undergoing dialysis treatment, presenting with edema or unable to perform proposed tests (gait speed and handgrip strength - HGS) were excluded.

Assuming alpha and beta errors of 5% and 20%, respectively, 0.4 (p) correlation between muscle mass and length of hospital stay (based on a preliminary pilot study), and 0.15 variability (d²), the minimum sample size was set at 84 individuals. This number was increased by 20% (n=101 patients) to offset occasional losses.

### Sarcopenia

Patients were assessed for sarcopenia within 48 hours of admission. Sarcopenia diagnosis was based on muscle mass, muscle strength and physical performance; sarcopenic patients were defined as those presenting with loss of muscle mass and concurrent decrease in muscle strength or physical performance. Pre-sarcopenia and severe sarcopenia were also accounted for. Pre-sarcopenia was defined as unfavorable lean mass assessment findings in non-sarcopenic patients (*i.e*., a stage preceding sarcopenia). Severe sarcopenia was defined according to three combined criteria: loss of muscle mass, loss of muscle strength and poor physical performance.^(^^3^^)^

### Muscle mass

Muscle mass estimates were based on a skeletal muscle mass index (SMMI), calculated using the following equation: $\text{SMMI}=\text{SMM}/{\text{height}}^{2}$. Skeletal muscle mass (SMM) was calculated according to Janssen et al.:^(^^8^^)^$\text{SMM}0(\text{kg})=[({\text{height}}^{2}/\text{resistance}\times \text{0}.401)+(\text{sex}\times 3.825)+(\text{age}\times \text{-0}.071)]+5.102$(height expressed in centimeters; resistance expressed in ohms; 1 and zero = male and female gender respectively; age given in years).

Muscle mass assessment criteria were based on SMMI cutoff values determined in NHANES III.^(^^9^^)^ In that study,^(^^9^^)^ 4,449 individuals aged ≥60 years were evaluated and SMMI <6.76kg/m² and <10.76kg/m² established as references for low muscle mass in women and men, respectively.^(^^10^^)^ Resistance was determined by bioelectrical impedance analysis (BIA) performed using portable equipment (Biodynamics, model 310).

### Muscle strength

Muscle strength was estimated from HGS measurements made using a digital dynamometer (JAMAR^®^). Cutoff values were determined according to HGS classification criteria proposed by Lauretani et al.,^(^^11^^)^ where HGS <30kg/f and <20kg/f, reflect unfavorable values for women and men, respectively.

### Physical performance

Physical performance assessment was based on the gait speed test (walking speed timed over a predetermined 4-meter trajectory on a level surface); low and normal gait speeds were defined as < and >0.8 meters/second, respectively. The fastest out of two repetitions was used in the analysis.^(^^12^^)^

### Sarcopenic obesity

Sarcopenic obesity was defined as abdominal circumference (AC) ≥88cm for women and ≥102cm for men, combined with a diagnosis of sarcopenia.^(^^13^^)^

Abdominal circumference was measured using a non-elastic measuring tape placed midway between the last rib and the iliac crest, and read to the nearest 0.1cm.^(^^14^^)^ Bony landmarks were located and palpated by the examiner at the level of the axillary midline. The measuring tape was wrapped around the abdomen in the horizontal plane and kept strictly parallel to the ground. Measurements were made at end of a normal expiration with patients standing upright; the tape was kept close to the skin and no pressure applied. Two measurements were obtained per landmark.^(^^15^^,^^16^^)^

### Cardiovascular risk and prognostic markers

The following variables were evaluated: troponin and creatine kinase MB isoenzyme (CKMB) values, acute myocardial infarction (AMI) classification according to ST segment elevation (ST segment elevation myocardial infarction, STEMI, or non-ST segment elevation myocardial infarction, NSTEMI), need for coronary angioplasty or myocardial revascularization, complications during hospital stay (infections, intensive care unit/ICU stay and/or death), length of hospital stay (days), readmission to the same unit (within 2 years of data collection, up to July 2017), thrombolysis in myocardial infarction (TIMI) scores reflecting risk of post-infarction complications (low, intermediate or high; zero to 2, 3 to 5, and >5, respectively) and mortality.^(^^17^^)^ Comorbidities such as *diabetes mellitus* (DM) and hypertension (HTN) were also accounted for. C-reactive protein (CRP) >5.0mg/dL was selected among inflammatory markers associated with CVR. Clinical data were extracted from medical records.

### Sociodemographic variables

The sociodemographic profile of the study population was delineated based on the following parameters: age, sex, race and level of education (years). Race/skincolor (white, brown or black)^(^^18^^)^ was determined by interviewers, then dichotomized into white and non-white for analytical purposes.

### Nutritional status

Nutritional status was determined according to body mass index (BMI, body weight in kilograms divided by the square of height in meters) and the cutoff points proposed by Lipschitz,^(^^19^^)^ (<22kg/m², 22 to 27kg/m² and >27kg/m², underweight, eutrophic and overweight, respectively) were adopted.

### Statistical analysis

Data tabulation and analysis were carried out using Excel 2007 and the software Statistical Package for the Social Sciences (SPSS), version 13.0 (Chicago, IL, USA). Descriptive analysis of variables consisted of calculation of frequency distributions and measures of central tendency. Continuous variables were tested for normality using the Kolmogorov-Smirnov test. Normally distributed variables were expressed as mean and standard deviation, and analyzed using the parametric Student's *t* test. Non-normally distributed variables (CKMB, troponin values and TIMI scores), were expressed as median and interquartile ranges, and analyzed using the non-parametric Mann Whitney U-test. Associations between categorical variables were investigated using Pearson's χ^2^ test. The level of significance was set at 5% (p<0.05).

### Ethical aspects

This study was approved by the Research Ethics Committee (CEP) for projects involving human beings of *Hospital Universitário Oswaldo Cruz/Pronto-Socorro Cardiológico Universitário de Pernambuco Prof. Luiz Tavares,* in compliance with resolution 466/12 of *Conselho Nacional de Saúde/Ministério da Saúde* [National Health Council/Ministry of Health], no. 980.370, CAAE: 41990815.6.00005192. Participants were duly informed of objectives of the study, data collected and potential risks and benefits derived from participation in the study. Volunteer participants signed an Informed Consent Form (ICF).

## RESULTS

The final sample comprised 99 patients with AMI, after exclusions due to inconsistent data. Mean age was 71.6 (±7.4) years; male and female patients were equally represented and non-white individuals prevailed (68.7%). Prevalence of hypertension and DM corresponded to 90.9% and 45.5%, respectively. Elevated CRP was detected in 67.4% of individuals and NSTEMI prevailed in the sample (73.2%). Mortality was 5.1%, 13.3% of patients required intensive care and 2.0% developed infections. Prevalence of malnourishment was 11.1%, whereas excess weight and abdominal obesity were detected in 41.4% and 52.4% of sample, respectively. Intermediate to high TIMI scores were attributed to 76% of the study population (Table 1).

**Table 1:** Sociodemographic and clinical features of elderly patients with coronary heart disease hospitalized at a cardiology reference center in the Northeastern region of Brazil

Variable		n (%)
Sex		
	Male	49 (49.5)
	Female	50 (50.5)
Age, years		
	60-69	42 (42.4)
	70-79	42 (42.4)
	≥80	115 (15.2)
Race		
	White	31 (31.3)
	Non-white	68 (68.7)
Hypertension		90 (90.9)
*Diabetes mellitus*		45 (45.5)
Nutritional status		
	Underweight	11 (11.1)
	Eutrophic	47 (47.5)
	Overweight	41 (41.4)
	Abdominal obesity	52 (52.4)
CRP		
	Normal	29 (32.6)
	Elevated	60 (67.4)
AMI therapy		
	Clinical management	56 (57.1)
	Angioplasty	17 (17.3)
	Revascularization surgery	25 (25.5)
Complications during hospital stay		
	Yes	20 (20.4)
	No	78 (79.6)
AMI classification		
	STEMI	25 (25.8)
	NSTEMI	71 (73.2)
Readmission		
	Yes	17 (17.2)
	No	82 (82.8)
TIMI		
	Low risk	23 (24.0)
	Intermediate risk	56 (58.3)
	High risk	17 (17.7)

CRP: C-reactive protein; AMI: acute myocardial infarction; STEMI: ST elevation acute myocardial infarction; NSTEMI: non-ST elevation acute myocardial infarction; TIMI: thrombolysis in myocardial infarction.

Prevalence of sarcopenia was 64.6%, with 70.3% of severely sarcopenic patients. Pre-sarcopenia was diagnosed in 22.9% of non-sarcopenic patients. As regards components of the diagnostic criteria for sarcopenia, physical performance was the most severely compromised (84.3%), followed by low muscle mass and low muscle strength (73.7% and 63.6%, respectively). Approximately 35% of patients were diagnosed with SO (Table 2).

**Table 2:** Characteristics of sarcopenia and sarcopenic obesity in elderly patients with coronary heart disease hospitalized at a cardiology reference center in the Northeastern region of Brazil

Variable		n (%)
Sarcopenia		
	Yes	64 (64.6)
	No	35 (35.4)
Sarcopenia classification		
	Sarcopenia	19 (29.7)
	Severe sarcopenia	45 (70.3)
Classification of non-sarcopenic individuals		
	Non-sarcopenic	27 (77.1)
	Pre-sarcopenic	8 (22.9)
Diagnostic components of sarcopenia		
	Reduced muscle mass*	73 (73.7)
	Reduced muscle strength†	63 (63.6)
	Low physical perfomance‡	75 (84.3)
Sarcopenic obesity		
	Yes	35 (35.4)
	No	62 (62.6)

* <6.76kg/m² for women and <10.76kg/m² for men;

† <30kg/f for men and <20kg/f for women;

‡ <0.8 meters/second.

Analysis of associations between sarcopenia and CVR variables revealed higher prevalence of sarcopenia among male patients (76.0% and 53.1%, male and female patients, respectively); p=0.017) and increasing prevalence with age (93.3% prevalence in individuals aged 80 years or older, p=0.008). Thrombolysis in myocardial infarction score was the only CVR marker significantly associated with sarcopenia, with higher median scores attributed to sarcopenic individuals (5.0, IQR: 3.0-5.0 *versus* 3.0, IQR: 2.0-4.0; p=0.002) (Table 3).

**Table 3:** Associations among sarcopenia, sociodemographic and clinical variables, and prognostic markers of coronary heart disease risk in hospitalized elderly patients with coronary heart disease

Variable		Sarcopenia		No sarcopenia		p value*
		n	%	n	%	
Sex						0.017
	Male	38	76.0	12	24.0	
	Female	26	53.1	23	46.9	
Age group, years						0.008
	60-69	21	50.0	21	50.0	
	70-79	29	69.0	13	31.0	
	≥80	4	93.3	1	6.7	
Hypertension						0.602
	Yes	58	64.4	32	35.6	
	No	6	66.7	3	33.3	
*Diabetes mellitus*						0.969
	Yes	29	64.4	16	35.6	
	No	35	64.8	19	35.2	
CRP						0.971
	Normal	18	62.1	11	37.9	
	Elevated	37	61.7	23	38.3	
AMI therapy						0.502
	Clinical management	35	62.5	21	37.5	
	Angioplasty	13	76.5	4	23.5	
	Revascularization surgery	15	60.0	10	40.0	
Complications during hospital stay						0.550
	Yes	14	70.0	6	30.0	
	No	49	62.8	29	37.2	
AMI classification						0.165
	STEMI	19	76.0	6	24.0	
	NSTEMI	43	60.6	28	39.4	
Readmission						0.581
	Yes	10	58.8	7	41.2	
	No	54	65.9	28	34.1	

Variable		Sarcopenia		No sarcopenia		p value†
		Median	IQR	Median	IQR	
Troponin		0.11	0.03-0.97	0.17	0.01-0.58	0.342
CKMB		6.67	2.1-25.0	5.5	1.7-13.3	0.555
TIMI		5.0	3.0-5.0	3.0	2.0-4.0	0.002

Variable		Mean	SD	Mean	SD	p value‡
Length of hospital stay		19.3	10.2	19.7	13.3	0.866

* χ^2^;

† Mann-Whitney U-test;

‡ Student's *t* test.

CRP: C-reactive protein; AMI: acute myocardial infarction; STEMI: ST elevation acute myocardial infarction; NSTEMI: non-ST elevation acute myocardial infarction; IQR: interquartile range; CKMB: creatine kinase MB isoenzyme; TIMI: thrombolysis in myocardial infarction; SD: standard deviation.

Sarcopenic obesity was not associated with demographic variables or CVR (Table 4).

**Table 4:** Associations among sarcopenic obesity, sociodemographic and clinical variables, and prognostic markers of coronary heart disease risk in hospitalized elderly patients with coronary heart disease

Variable		Sarcopenic obesity		No sarcopenic obesity		p value*
		n	%	n	%	
Sex						0.120
	Male	21	43.8	27	56.3	
	Female	14	28.6	35	71.4	
Age, years						0.398
	60-69	12	28.6	30	71.4	
	70-79	17	42.5	23	57.5	
	≥80	6	40.0	9	60.0	
Hypertension						0.150
	Yes	34	38.6	54	61.4	
	No	1	11.1	8	88.9	
*Diabetes mellitus*						0.290
	Yes	18	41.9	25	58.1	
	No	17	31.5	37	68.5	
CRP						0.074
	Normal	13	44.8	16	55.2	
	Elevated	15	25.9	43	74.1	
AMI therapy						0.776
	Clinical management	20	37.0	34	63.0	
	Angioplasty	5	29.4	12	70.6	
	Revascularization surgery	10	40.0	15	60.0	
Complications during hospital stay						0.500
	Yes	6	30.0	14	70.0	
	No	29	38.2	47	61.8	
AMI classification						0.882
	STEMI	9	36.7	16	64.0	
	NSTEMI	26	37.7	43	62.3	
Readmission						0.183
	Yes	4	23.5	13	76.5	
	No	31	38.8	49	61.3	

Variable		Sarcopenic obesity		No sarcopenic obesity		p value†
		Median	IQR	Median	IQR	
Troponin		0.06	0.02-0.86	0.20	0.04-0.79	0.181
CKMB		4.2	1.4-17.4	8.3	2.5-19.7	0.343
TIMI		4.0	2.0-6.0	4.0	3.0-5.0	0.655

Variable		Mean	SD	Mean	SD	p value‡
Length of hospital stay		20.2	11.6	19.2	11.1	0.669

* χ^2^;

† Mann-Whitney U-test;

‡ Student's *t* test.

CRP: C-reactive protein; AMI: acute myocardial infarction; STEMI: ST elevation acute myocardial infarction; NSTEMI: non-ST elevation acute myocardial infarction; IQR: interquartile range; CKMB: creatine kinase MB isoenzyme; TIMI: thrombolysis in myocardial infarction; SD: standard deviation.

## DISCUSSION

Exponential growth of the elderly population has led to increased interest in investigation of age-related complications globally. Sarcopenia and SO are thought to be important risk factors for age-related adverse events, such as increased risk of falls and fractures, higher hospital admission rates and higher mortality. Also, there are evidences to suggest a relation with CVD,^(^^20^^–^^22^^)^ in spite of the scarcity of studies analyzing the role of these conditions as risk potentiating factors in patients with established CVD. Are there reasons to suspect associations between sarcopenia or SO and increased risk of complications in patients with coronary heart disease?

The sample in this study comprised elderly patients with high CVR, hospitalized due to coronary events and with high prevalence of HTN, DM, excess body weight and abdominal obesity. A significant percentage of these patients (76%) had intermediate to high CVR according to post-infarction risk scores TIMI, a sensitive tool for detection of coronary heart disease-related complications. Hence, findings of this study must be interpreted in the light of sample characteristics.

High prevalence of sarcopenia (64.4%) emphasizes the significance of sarcopenia assessment in hospitalized elderly patients with coronary heart disease. Previous investigations suggest the prevalence of sarcopenia in these patients may exceed 50%, as shown by Baumgartner et al.,^(^^1^^)^ in a study evaluating Hispanic and non-Hispanic elderly patients with a mean age of 73.6 years, and listed in the health system of New Mexico, USA. A second study based on data of 1,578 individuals aged over 65 years extracted from The Korea National Health and Nutrition Examination Study (KNHANES IV) reported sarcopenia in 30.3% and 29.3% of males and females, respectively. In that study, body composition was determined using dual-energy X-ray absorptiometry (DEXA).^(^^21^^)^ Disparities may be attributed to different methods employed to diagnose sarcopenia between studies, or reflect ethnicity-related differences.

According to recent studies, sarcopenia is a risk factor for cardiovascular and/or metabolic complications. In the study by Chin et al.,^(^^21^^)^ sarcopenia was considered an independent risk factor, given the higher prevalence of CVD in sarcopenic compared to non-sarcopenic patients (p=0.008). Independent associations between sarcopenia and CVR have also been reported by Han et al.,^(^^23^^)^ in a study involving a Chinese population. According to authors of that study,^(^^23^^)^ DM and HTN would be major factors associated with sarcopenia, as DM promotes loss of muscle mass and strength in response to hyperglycemia, insulin resistance, endocrine changes and release of inflammatory cytokines. Chronic hyperglycemia leads to increased levels of advanced glycation end-products; these, in turn, accumulate in skeletal muscle and cartilage tissues, increasing muscle stiffness in diabetic patients. Also, inflammatory cytokines, such as tumor necrosis factor and interleukin 6, present in both DM and HTN, have negative impacts on muscle mass and strength and physical performance in older adults.

According to Han et al.,^(^^23^^)^ DM and HTN affect muscle mass, muscle strength and physical performance, leading to sarcopenia; however, further studies are needed to elucidate this relation. Lack of associations between DM or HTN and sarcopenia in this study should be emphasized and suggests mechanisms other than DM and HTN may interfere with muscle mass retention and functional capacities.

Severe sarcopenia was detected in 70.3% of sarcopenic patients in this sample. Severe sarcopenia combines loss of muscle mass and strength and functional compromise, increasing the risk of undesirable outcomes, such as higher morbidity and mortality, lower quality of life and higher hospital costs.^(^^3^^)^ These findings underscore data derived from studies evaluating elderly patients seen at the general geriatric outpatient clinic of a university hospital, located in the Northeastern region of Brazil, which revealed 66.7% prevalence of severe sarcopenia.^(^^24^^)^ In contrast, in a study carried out by Smoliner et al.,^(^^25^^)^ at a German hospital, only 25.3% of patients were diagnosed sarcopenia, and the prevalence of severe sarcopenia in this group was low (18.7%). In that study,^(^^25^^)^ the sample comprised elderly individuals hospitalized due to general acute conditions and similar criteria to this investigation were employed.

The percentage of sarcopenic obese individuals in this sample (35%) is higher compared to available data on cardiac patients, with most studies reporting less than 10% prevalence.^(^^26^^–^^28^^)^ However, prevalence may vary widely (0.41%) according to study population and methods employed for definition.^(^^29^^)^ Sarcopenic obesity was not associated with coronary heart disease risk parameters in this study. Still, potential relations have been suggested.

Aging in associated with decreased levels of physical activity, which, in turn, leads to loss of muscle mass and strength, promoting still lower levels of physical activity and resistance.^(^^3^^,^^30^^,^^31^^)^ Poor muscle quality with greater fatty infiltration may contribute to higher levels of inflammation. Lower levels of physical activity may lead to weight gain, increased insulin resistance and higher total abdominal fat, with concurrent rise in inflammation levels. Increased production of leptin and other adipokines and cytokines by fat tissue mass may contribute to SO development. The vicious cycle between lean mass loss and fat mass gain may culminate in sarcopenia and SO.^(^^29^^)^

Likewise other studies reporting greater muscle mass in men compared to women, sarcopenia was more pronounced in male and older individuals in this sample. Similar findings have been described by Janssen et al.,^(^^10^^)^ in a study reporting 31% and 64% prevalence of sarcopenia in older women and men, respectively. This phenomenon reflects greater loss of muscle mass in response to declining levels of growth hormone, insulin-related growth factor (IGF-1) and testosterone in men; also, men tend to adapt poorly to muscle tissue loss compared to women.^(^^31^^,^^32^^)^ Increasing sarcopenia with age is also associated with age-related morphological and functional changes, such as decreased fat-free mass and muscle strength, which lead to sarcopenia.^(^^33^^,^^34^^)^

Associations between prognostic and coronary heart disease risk markers in this study were limited to TIMI scores, *i.e*., sarcopenic patients with coronary heart disease scored higher and were, therefore, at greater risk of complications compared to non-sarcopenic elderly patients. Similar analyses are lacking in literature and should be undertaken to corroborate these findings. In any case, sarcopenia is thought to occur concurrently with fat mass increase rather than as an isolated event.^(^^3^^)^ Lipid infiltration contributes to sarcopenia via macrophage-mediated release of pro-inflammatory cytokines and adipokines, which induce chronic inflammation. Sarcopenic individuals tend to have functional and physical impairments associated with lower levels of anti-inflammatory factors induced by muscle contraction − or “myokines”. Relative myokine scarcity in sarcopenia may increase CVD risk. Overall, functional decline and chronic inflammation, as well as lower levels of anti-inflammatory substances in sarcopenic individuals, promote insulin resistance, type 2 DM, hyperlipidemia and hypertension, and may eventually increase the risk of CVD.^(^^21^^)^ However, these findings must be interpreted with caution, bearing in mind that markers not associated with sarcopenia in this study have not been investigated to date.

Limitations of this study must be emphasized. Retrospective study design relying on medical records may have impacted the analysis due to incomplete or missing data. Relatively small sample size may have limited statistical power. Finally, single center sample comprising a specific group of coronary heart disease patients (AMI patients) limits extrapolation of data to other populations.

## CONCLUSION

Studies investigating sarcopenia and sarcopenic obesity as markers of coronary heart disease risk are still scarce. This study revealed high prevalence of sarcopenia among patients with coronary heart disease, particularly older male patients. Sarcopenic obesity was less common than sarcopenia; still approximately one-third of patients were affected. Thrombolysis in myocardial infarction score was the only prognostic and cardiovascular diseases risk marker associated with sarcopenia; sarcopenic obesity was not associated with any of the selected markers. Further studies are warranted to elucidate the potential value of sarcopenia and sarcopenic obesity as predictors of complications and prognosis in patients with coronary heart disease. In any case, these conditions are associated with adverse outcomes and should therefore be investigated in routine patient follow-up.
