# Supplementary material for: Sarcopenia and sarcopenic obesity as prognostic predictors in hospitalized elderly patients with acute myocardial infarction
Source: Einstein (Sao Paulo). 2019 Aug 14;17(4):eAO4632. doi: 10.31744/einstein_journal/2019AO4632 (PMC6706224; doi:10.31744/einstein_journal/2019AO4632)
Supplement: Translation

# Translation: Relação entre sarcopenia e obesidade sarcopênica como preditores de prognóstico em pacientes idosos hospitalizados com infarto agudo do miocárdio

**Authors:** Natália de Moraes Santana, Roberta Maria Lins Mendes, Nadja Fernandes da Silva, Cláudia Porto Sabino Pinho

**Affiliations:** 1 Universidade de Pernambuco, Recife, PE, Brasil

**Keywords:** Sarcopenia, Doenças cardiovasculares, Infarto do miocárdio, Obesidade, Idoso

## Abstract

**Objetivo:**

Verificar a relação entre sarcopenia e obesidade sarcopênica como preditores de prognóstico em pacientes idosos com infarto agudo do miocárdio internados.

**Métodos:**

Estudo transversal envolvendo pacientes idosos com infarto agudo do miocárdio, hospitalizados no período de abril a julho de 2015, em serviço público, no Nordeste brasileiro. A sarcopenia foi determinada por meio das medidas de massa muscular, força muscular e desempenho físico. Foram utilizados os marcadores de risco cardiovascular e de prognóstico, como os valores de troponina e da isoenzima MB da creatinina quinase, classificação do infarto agudo do miocárdio de acordo com a elevação do segmento ST e o escore de risco de trombólise em infarto do miocárdio.

**Resultados:**

Foram avaliados 99 pacientes, com média de idade de 71,6 (±7,4) anos. Verificou-se prevalência de sarcopenia de 64,6% e 35,4% de obesidade sarcopênica. A sarcopenia foi mais prevalente no sexo masculino (p=0,017), na faixa etária >80 anos (p=0,008). Dentre os marcadores de risco cardiovascular, apenas o escore de risco trombólise em infarto do miocárdio esteve estatisticamente associado à sarcopenia (p=0,002).

**Conclusão:**

A prevalência da sarcopenia foi elevada e se associou com o escore de risco de trombólise em infarto do miocárdio. A obesidade sarcopênica acometeu cerca de um terço dos pacientes e não se associou a nenhum parâmetro preditor prognóstico.

## INTRODUÇÃO

Sarcopenia é uma síndrome geriátrica, multifatorial, definida como a perda progressiva de massa muscular associada à redução da força muscular e/ou desempenho físico. Pode ter como consequências dificuldades motoras e comprometimento da autonomia do indivíduo acometido, resultando em menor qualidade de vida e maior risco de morbimortalidade.^(^^1^^–^^3^^)^ Os dados sobre a incidência e a prevalência de sarcopenia ainda são escassos na literatura nacional. A prevalência de sarcopenia varia de 5 a 50%,^(^^3^^)^ e essas variações entre as diferentes prevalências podem ser devido a diversos fatores, como diferenças na composição étnica da amostra, utilização de diferentes parâmetros para a definição de sarcopenia e/ou da adoção métodos variados de mensuração da massa magra.

Riscos metabólicos e cardiovasculares estão intimamente relacionados ao envelhecimento, e é crescente o interesse sobre os fatores que podem levar a seus distúrbios, visto que as doenças cardiovasculares (DCV) constituem uma das causas principais de incapacidade e morte em todo o mundo. Apesar de alguns estudos demonstrarem consistente associação entre baixa massa muscular e risco de mortalidade, a associação entre a sarcopenia e o risco de DCV ainda não está totalmente estabelecida.^(^^4^^,^^5^^)^

Em recente revisão, estudos clínicos observacionais demonstraram que a baixa massa muscular estaria associada à rigidez arterial, que é um preditor independente de DCV. Além disso, a sarcopenia teria potencial efeito no processo de aterogênese decorrente do aumento relativo do tecido adiposo secundário à perda de massa muscular e à substituição de miócitos por adipócitos.^(^^6^^)^ Dessa forma, a sarcopenia, na presença de excesso de tecido adiposo, caracterizando o quadro de obesidade sarcopênica (OS), teria um efeito ainda maior sobre doenças metabólicas, DCV e mortalidade, do que obesidade ou sarcopenia sozinhos.^(^^7^^)^

Com o crescimento mundial da população idosa, há necessidade de maior atenção das situações adversas que podem ser agravadas nesta faixa etária, como é o caso da sarcopenia e da OS, que estão diretamente relacionadas com a perda de funcionalidade e da autonomia e, possivelmente, ao pior prognóstico cardiovascular.

## OBJETIVO

Verificar a associação entre sarcopenia e obesidade sarcopênica como preditores de prognóstico em pacientes idosos com infarto agudo do miocárdio.

## MÉTODOS

Estudo retrospectivo desenvolvido em hospital público referência em cardiologia no Nordeste brasileiro com coleta de dados no período de abril a julho de 2015, e, posteriormente, com resgate de dados em prontuário após 2 anos desse primeiro internamento. A amostra foi constituída por pacientes de ambos os sexos, com idade ≥60 anos, internados em enfermaria de coronariopatias. Pacientes com limitações físicas (portadores de amputações) e cognitivas (sem condições de estabelecer contato com o entrevistador), restritos ao leito, no pós-operatório de cirurgias cardíacas, portadores de doença renal em tratamento dialítico, que apresentavam edema e impossibilitados de realizar os testes propostos (velocidade de marcha e força de preensão manual − FPM) foram excluídos do estudo.

Considerando-se um erro alfa de 5%, um erro beta de 20%, correlação entre a massa muscular e o tempo de internamento de 0,4 (p) (obtida em um estudo piloto preliminar) e variabilidade de 0,15 (d²), foi obtido tamanho amostral mínimo de 84 indivíduos. Para cobrir eventuais perdas, esse número foi aumentado em 20%, totalizando n amostral de 101 pacientes.

### Sarcopenia

A sarcopenia foi avaliada até 48 horas da admissão do paciente no hospital, sendo identificada pelos componentes massa muscular, força muscular e desempenho físico, sendo considerado sarcopênico quando os indivíduos apresentassem diminuição da massa muscular associada à redução da força muscular ou do desempenho físico. Foi considerada, ainda, a classificação de pré-sarcopenia e sarcopenia severa. Pré-sarcopenia foi considerado quando os indivíduos, mesmo não sarcopênicos, apresentaram resultados desfavoráveis na avaliação da massa magra, condição que caracteriza um estágio prévio à sarcopenia. Sarcopenia severa foi estabelecida quando ocorreu a conjunção dos três critérios: redução da massa, da força muscular e baixo desempenho físico.^(^^3^^)^

### Massa muscular

A massa muscular foi avaliada a partir do índice de massa muscular esquelética (IMME), obtido por meio da equação: $\text{IMME}=\text{MME}/{\text{altura}}^{2}$, onde MME corresponde a massa muscular esquelética. A MME, por sua vez, foi calculada de acordo com a fórmula de Janssen et al.:^(^^8^^)^$\text{MME}(\text{kg})=[({\text{altura}}^{2}/\text{resistência}\times \text{0},401)+(\text{sexo}\times 3,825)+(\text{idade}\times \text{-0},071)]+5,102$ , onde: altura em centímetros; a resistência em ohms; 1 corresponde ao sexo masculino e zero ao feminino; e idade em anos.

Como critério de avaliação da massa muscular, foi considerado o ponto de corte para o IMME obtido no NHANES III,^(^^9^^)^ estudo que avaliou 4.449 indivíduos com idade ≥60 anos e estabeleceu como baixa massa muscular IMME <6,76kg/m² para mulheres e IMME <10,76kg/m² para homens.^(^^10^^)^ A medida de resistência foi obtida a partir de bioimpedância elétrica (BIA), utilizando-se o equipamento portátil da marca *Biodynamics* modelo 310.

### Força muscular

A força muscular foi obtida a partir da FPM, mensurada com uso do dinamômetro digital JAMAR^®^. Os pontos de corte foram estabelecidos segundo o critério de classificação da FPM sugerido por Lauretani et al.,^(^^11^^)^ no qual valores de FPM <30kg/f para homens e FPM <20kg/f para mulheres são considerados desfavoráveis.

### Desempenho físico

Para a avaliação do desempenho físico, foi realizado o teste de velocidade da marcha, sendo considerada a velocidade de caminhada em um percurso de 4 metros previamente demarcado em superfície plana. Foi considerada marcha lenta quando a velocidade fosse <0,8 metro/segundo e normal quando fossem encontrados valores superiores a esse. Foi utilizada a mais rápida de duas repetições.^(^^12^^)^

### Obesidade sarcopênica

A OS foi considerada quando o indivíduo apresentou valores de circunferência abdominal (CA) ≥88cm para as mulheres e ≥102cm para os homens, associada ao diagnóstico de sarcopenia.^(^^13^^)^

A CA foi obtida com fita métrica inelástica, com precisão de 0,1cm, diretamente sobre o ponto médio entre a última costela e a crista ilíaca.^(^^14^^)^ Os marcos ósseos foram localizados e palpados pelo examinador ao nível da linha axilar média. A fita de medição foi colocada em um plano horizontal em torno do abdomen, e especial atenção foi dada para garantir que a fita estivesse paralela ao chão. A medição foi realizada no final da expiração normal com a fita inelástica adjacente à pele, mas sem comprimi-la, com o participante em pé e bem ereto. Para cada ponto antropométrico avaliado, uma dupla medição foi obtida.^(^^15^^,^^16^^)^

### Marcadores de risco cardiovascular e de prognóstico

Foram avaliadas as variáveis: valores de troponina e da isoenzima MB da creatinina quinase (CKMB), classificação do infarto agudo do miocárdio (IAM) de acordo com a elevação do segmento ST (IAM com ou sem supradesnivelamento do segmento ST, IAMCSST ou IAMSST, respectivamente), necessidade de angioplastia coronariana ou cirurgia de revascularização do miocárdio, complicações durante o internamento (infecções, passagem por unidade de terapia intensiva – UTI − e/ou óbito), tempo de internamento (em dias), ocorrência de reinternamento no mesmo serviço (no período de 2 anos após a coleta, até julho de 2017) e escore *thrombolysis in myocardial infarction* (TIMI), que avalia o risco de complicações pós-infarto (zero a 2: baixo risco; 3 a 5: risco médio; >5 alto risco) e mortalidade.^(^^17^^)^ Foi considerada, ainda, a presença das comorbidades *diabetes mellitus* (DM) e hipertensão arterial sistêmica (HAS). O marcador de inflamação relacionado ao risco cardiovascular (RCV) utilizado foi a proteína C-reativa (PCR), considerando-se alterada quando >5,0mg/dL. Todos os dados clínicos foram resgatados do prontuário do paciente.

### Variáveis sociodemográficas

Para caracterização sociodemográfica da população estudada foram coletadas informações referentes a idade, sexo, raça e escolaridade (em anos de estudo). A raça/cor foi determinada pelo entrevistador e classificada como branca, parda e negra^(^^18^^)^ e, para fins analítico, posteriormente, foi dicotomizada em branco e não branco.

### Estado nutricional

O estado nutricional foi determinado segundo o índice de massa corporal (IMC) obtido a partir do quociente do peso (kg) pela altura (m) ao quadrado, sendo utilizado o ponto de corte proposto por Lipschitz,^(^^19^^)^ que considera <22kg/m² magreza, entre 22 e 27kg/m² eutrofia e >27kg/m² excesso de peso.

### Análise estatística

A tabulação e a análise dos dados foram realizadas no programa Excel 2007 e do pacote estatístico *Statistical Package for the Social Sciences* (SPSS), versão 13.0 (Chicago, IL, EUA). Foi realizada análise descritiva das variáveis mediante cálculo das distribuições de frequência e medidas de tendência central. As variáveis contínuas foram testadas segundo a normalidade de distribuição pelo teste de Kolmogorov-Smirnov. Quando apresentaram distribuição normal foram descritas na forma de média e desvio padrão e o respectivo teste paramétrico foi aplicado (*t* de Student). Quando apresentaram distribuição não normal (CKMB, troponina e TIMI), foram descritas na forma de mediana e intervalo interquartílico, e o teste não paramétrico “U” de Mann Whitney foi empregado. A associação entre as variáveis categóricas foi analisada por meio do teste do χ^2^ de Pearson. O nível de significância adotado para todos os testes foi menor que 0,05.

### Aspectos éticos

A pesquisa teve aprovação do Comitê de Ética em Pesquisa (CEP) envolvendo seres humanos do complexo hospitalar Hospital Universitário Oswaldo Cruz/Pronto-Socorro Cardiológico Universitário de Pernambuco Prof. Luiz Tavares, de acordo com a resolução 466/12 do Conselho Nacional de Saúde/Ministério da Saúde, sob o número de 980.370, CAAE: 41990815.6.00005192. Todos os procedimentos adotados foram explicados aos pacientes, assim como os objetivos do estudo, os parâmetros coletados, bem como possíveis riscos e benefícios de sua participação. Os que concordaram em participar assinaram o Termo de Consentimento Livre e Esclarecido (TCLE).

## RESULTADOS

Após eliminadas as perdas por inconsistência de informações, 99 pacientes com IAM foram efetivamente analisados. A média de idade foi 71,6 (±7,4) anos, com distribuição equitativa entre os sexos e predomínio de indivíduos não brancos (68,7%). A prevalência de hipertensão foi 90,9% e do DM foi 45,5%. A PCR foi elevada em 67,4% dos indivíduos, e o IAMSST predominou na amostra (73,2%). A mortalidade foi de 5,1%, a passagem por UTI foi observada em 13,3% e a ocorrência de infecções em 2,0%. A prevalência de desnutrição foi 11,1%, enquanto o excesso de peso e a obesidade abdominal foram encontrados em 41,4% e 52,4% da amostra, respectivamente. O médio e alto risco do TIMI juntos estiveram presentes em 76% da população estudada (Tabela 1).

**Tabela 1:** Características sociodemográficas e clínicas dos pacientes idosos coronariopatas internados em hospital referência em cardiologia no Nordeste brasileiro

Variável		n (%)
Sexo		
	Masculino	49 (49,5)
	Feminino	50 (50,5)
Faixa etária, anos		
	60-69	42 (42,4)
	70-79	42 (42,4)
	≥80	115 (15,2)
Raça		
	Branco	31 (31,3)
	Não branco	68 (68,7)
Hipertensão		90 (90,9)
*Diabetes mellitus*		45 (45,5)
Estado nutricional		
	Magreza	11 (11,1)
	Eutrofia	47 (47,5)
	Excesso de peso	41 (41,4)
	Obesidade abdominal	52 (52,4)
PCR		
	Normal	29 (32,6)
	Elevada	60 (67,4)
Terapia do IAM		
	Tratamento clínico	56 (57,1)
	Angioplastia	17 (17,3)
	Cirurgia de revascularização	25 (25,5)
Complicações no internamento		
	Sim	20 (20,4)
	Não	78 (79,6)
Classificação do IAM		
	IAMCSST	25 (25,8)
	IAMSST	71 (73,2)
Internamento posterior		
	Sim	17 (17,2)
	Não	82 (82,8)
TIMI		
	Baixo risco	23 (24,0)
	Médio risco	56 (58,3)
	Alto risco	17 (17,7)

PCR: proteína C-reativa; IAM: infarto agudo do miocárdio; IAMCSST: infarto agudo do miocárdio com supradesnivelamento do segmento ST; IAMSST: infarto agudo do miocárdio sem supradesnivelamento do segmento ST; TIMI: *thrombolysis in myocardial infarction.*

A prevalência de sarcopenia foi 64,6%, dentre os quais 70,3% foram classificados como sarcopênico severo. Dentre os não sarcopênicos, 22,9% apresentaram pré-sarcopenia. Dentre os componentes utilizados no critério diagnóstico da sarcopenia, o desempenho físico foi o mais comprometido (84,3%), seguido da baixa massa muscular (73,7%) e da força muscular (63,6%). Cerca de 35% dos indivíduos apresentaram diagnóstico de OS (Tabela 2).

**Tabela 2:** Magnitude da sarcopenia e obesidade sarcopênica em pacientes coronariopatas idosos internados em hospital referência em cardiologia no Nordeste brasileiro

Variável		n (%)
Sarcopenia		
	Sim	64 (64,6)
	Não	35 (35,4)
Classificação da sarcopenia		
	Sarcopenia	19 (29,7)
	Sarcopenia severa	45 (70,3)
Classificação dos não sarcopênicos		
	Sem sarcopenia	27 (77,1)
	Pré-sarcopenia	8 (22,9)
Componentes diagnósticos da sarcopenia		
	Massa muscular reduzida*	73 (73,7)
	Força muscular reduzida†	63 (63,6)
	Baixo desempenho físico‡	75 (84,3)
Obesidade sarcopênica		
	Sim	35 (35,4)
	Não	62 (62,6)

* <6,76kg/m² para mulheres e <10,76kg/m² para homens;

† <30kg/f para homens e <20kg/f para mulheres;

‡ <0,8 metros/segundo.

Na análise da associação da sarcopenia com as variáveis de RCV, verificou-se que a sarcopenia foi mais prevalente no sexo masculino (76,0% e 53,1%, homens e mulheres, respectivamente; p=0,017) e que aumentou com a progressão da idade, com prevalência de 93,3% nos indivíduos com idade igual ou superior a 80 anos (p=0,008). Dentre os marcadores de RCV, apenas o escore TIMI foi estatisticamente associado à sarcopenia, com mediana do escore mais elevada nos indivíduos sarcopênicos (5,0 (IIQ: 3,0-5,0) *versus* 3,0 (IIQ: 2,0-4,0); p=0,002) (Tabela 3).

**Tabela 3:** Associação de sarcopenia com variáveis sociodemográficas, clínicas e marcadores de prognóstico de risco de doença coronariana em pacientes coronariopatas idosos hospitalizados

Variável		Sarcopenia		Sem sarcopenia		Valor de p*
		n	%	n	%	
Sexo						0,017
	Masculino	38	76,0	12	24,0	
	Feminino	26	53,1	23	46,9	
Faixa etária, anos						0,008
	60-69	21	50,0	21	50,0	
	70-79	29	69,0	13	31,0	
	≥80	4	93,3	1	6,7	
Hipertensão arterial						0,602
	Sim	58	64,4	32	35,6	
	Não	6	66,7	3	33,3	
*Diabetes mellitus*						0,969
	Sim	29	64,4	16	35,6	
	Não	35	64,8	19	35,2	
PCR						0,971
	Normal	18	62,1	11	37,9	
	Elevada	37	61,7	23	38,3	
Terapia do IAM						0,502
	Tratamento clínico	35	62,5	21	37,5	
	Angioplastia	13	76,5	4	23,5	
	Cirurgia de revascularização	15	60,0	10	40,0	
Complicações no internamento						0,550
	Sim	14	70,0	6	30,0	
	Não	49	62,8	29	37,2	
Classificação do IAM						0,165
	IAMCSST	19	76,0	6	24,0	
	IAMSST	43	60,6	28	39,4	
Internamento posterior						0,581
	Sim	10	58,8	7	41,2	
	Não	54	65,9	28	34,1	

Variável		Sarcopenia		Sem sarcopenia		Valor de p†
		Mediana	IIQ	Mediana	IIQ	
Troponina		0,11	0,03-0,97	0,17	0,01-0,58	0,342
CKMB		6,67	2,1-25,0	5,5	1,7-13,3	0,555
TIMI		5,0	3,0-5,0	3,0	2,0-4,0	0,002

Variável		Média	DP	Média	DP	Valor de p‡
Tempo de internamento		19,3	10,2	19,7	13,3	0,866

* χ^2^;

† U de Mann-Whitney;

‡ teste *t* de Student.

PCR: proteína C-reativa; IAM: infarto agudo do miocárdio; IAMCSST: infarto agudo do miocárdio com supradesnivelamento do segmento ST; IAMSST: infarto agudo do miocárdio sem supradesnivelamento do segmento ST; IIQ: intervalo interquartílico; CKMB: isoenzima MB da creatinina quinase; TIMI: *thrombolysis in myocardial infarction;* DP: desvio padrão.

A OS não se associou com as variáveis demográficas e do RCV (Tabela 4).

**Tabela 4:** Associação de obesidade sarcopênica com variáveis sociodemográficas, clínicas e marcadores de prognóstico de risco de doença coronariana em pacientes coronariopatas idosos hospitalizados

Variável		Obesidade sarcopênica		Sem obesidade sarcopênica		Valor de p*
		n	%	n	%	
Sexo						0,120
	Masculino	21	43,8	27	56,3	
	Feminino	14	28,6	35	71,4	
Faixa etária, anos						0,398
	60-69	12	28,6	30	71,4	
	70-79	17	42,5	23	57,5	
	≥80	6	40,0	9	60,0	
Hipertensão arterial						0,150
	Sim	34	38,6	54	61,4	
	Não	1	11,1	8	88,9	
*Diabetes mellitus*						0,290
	Sim	18	41,9	25	58,1	
	Não	17	31,5	37	68,5	
PCR						0,074
	Normal	13	44,8	16	55,2	
	Elevada	15	25,9	43	74,1	
Terapia do IAM						0,776
	Tratamento clínico	20	37,0	34	63,0	
	Angioplastia	5	29,4	12	70,6	
	Cirurgia de revascularização	10	40,0	15	60,0	
Complicações no internamento						0,500
	Sim	6	30,0	14	70,0	
	Não	29	38,2	47	61,8	
Classificação do IAM						0,882
	IAMCSST	9	36,7	16	64,0	
	IAMSST	26	37,7	43	62,3	
Internamento posterior						0,183
	Sim	4	23,5	13	76,5	
	Não	31	38,8	49	61,3	

Variável		Obesidade sarcopênica		Sem obesidade sarcopênica		Valor de p†
		Mediana	IIQ	Mediana	IIQ	
Troponina		0,06	0,02-0,86	0,20	0,04-0,79	0,181
CKMB		4,2	1,4-17,4	8,3	2,5-19,7	0,343
TIMI		4,0	2,0-6,0	4,0	3,0-5,0	0,655

Variável		Média	DP	Média	DP	Valor de p‡
Tempo de internamento		20,2	11,6	19,2	11,1	0,669

* χ^2^;

† U de Mann-Whitney;

‡ teste *t* de Student.

PCR: proteína C-reativa; IAM: infarto agudo do miocárdio; IAMCSST: infarto agudo do miocárdio com supradesnivelamento do segmento ST; IAMSST: infarto agudo do miocárdio sem supradesnivelamento do segmento ST; IIQ: intervalo interquartílico; CKMB: isoenzima MB da creatinina quinase; TIMI: *thrombolysis in myocardial infarction;* DP: desvio padrão.

## DISCUSSÃO

O crescimento exponencial da população idosa tem despertado interesse em todo o mundo para investigar as complicações que podem ocorrer nesta fase da vida. A sarcopenia e a OS têm sido indicadas como fatores de risco importantes para eventos adversos no envelhecimento, como maior risco de quedas e fraturas, maior número de hospitalizações e maior mortalidade. Além disso, evidências sugerem relação com as DCV,^(^^20^^–^^22^^)^ mas ainda há escassez de dados quando se trata da análise dessas condições como fatores potencializadores de risco em pacientes com DCV estabelecida. Será que a sarcopenia ou OS, em pacientes cardiopatas, estaria associada ao maior risco de complicações?

A população estudada é composta por pacientes idosos com alto RCV, avaliada em internamento por evento coronariano, marcada por elevada prevalência de HAS, DM, excesso de peso e obesidade abdominal, com percentual significativo indicando médio e alto RCV (76%), segundo o escore de risco pós-infarto TIMI, que é um marcador sensível para detectar o risco para complicações relacionadas às coronariopatias. Assim, os achados dessa investigação devem ser interpretados levando-se em conta essas características da amostra.

A elevada prevalência de sarcopenia (64,4%) demonstra a importância da avaliação dessa condição no idoso cardiopata hospitalizado. Achados de outras investigações indicam que a prevalência de sarcopenia em cardiopatas pode ser superior a 50%, como demonstrado por Baumgartner et al.,^(^^1^^)^ que avaliaram pacientes idosos com média de idade de 73,6 anos, hispânicos e não hispânicos, listados no sistema de saúde da cidade do Novo México, nos Estados Unidos. Outro estudo, que analisou dados do *The Korea National Health and Nutrition Examination Study* (KNHANES IV), envolvendo 1.578 pessoas acima de 65 anos, reportou que a sarcopenia esteve presente em 30,3% dos homens e 29,3% em mulheres, obtendo a composição corporal por meio da *dual-energy X-ray absorptiometry* (DEXA).^(^^21^^)^ Esta variação nos percentuais pode ser atribuída aos diferentes métodos utilizados para diagnosticar a sarcopenia, bem como diferentes etnias estudadas.

Estudos recentes apontam a sarcopenia como fator de risco para o desenvolvimento de complicações cardiovasculares e/ou metabólicas. Chin et al.,^(^^21^^)^ apontaram a sarcopenia como fator de risco independente, indicando que os indivíduos sarcopênicos apresentaram maior prevalência de DCV quando comparados aos não sarcopênicos (p=0,008). Ao investigar uma população chinesa, Han et al.,^(^^23^^)^ também encontraram associação independente entre sarcopenia e RCV, acrescentando que o DM e a HAS seriam os principais fatores associados à sarcopenia, pois o DM acelera a perda de massa e a força muscular devido à hiperglicemia, à resistência à insulina, às alterações endócrinas e à liberação de citocinas inflamatórias. A hiperglicemia crônica aumenta os produtos finais de glicosilação avançada; estes se acumulam no músculo esquelético e na cartilagem, e aumentam a rigidez muscular em pacientes com diabetes. Além disso, as citocinas inflamatórias, como o fator de necrose tumoral e a interleucina 6, presentes tanto no DM como na HAS, têm efeitos prejudiciais na massa muscular, na força e no desempenho físico em adultos mais velhos.

Han et al.,^(^^23^^)^ por sua vez, concluíram que o DM e a HAS afetam a massa muscular, a força e o desempenho físico, levando à sarcopenia, mas sugerem que mais estudos sejam realizados para elucidar essa relação. É importante destacar que nossos resultados não indicaram associação entre o DM e HAS com a sarcopenia. Portanto, é possível que outros mecanismos, além dessas duas condições, afetem a preservação muscular e funcionalidade.

Além disso, 70,3% dos sarcopênicos apresentaram grau severo da condição, na qual coexiste o comprometimento de massa muscular, força e funcionalidade, e, consequentemente, maior risco de desfechos indesejados, como maior morbimortalidade, declínio na qualidade de vida e aumento de custos hospitalares.^(^^3^^)^ Esse resultado é um dado que corrobora os dados apresentados em investigação que avaliou idosos atendidos no ambulatório geriátrico geral de um hospital universitário no Nordeste do Brasil, cuja prevalência de sarcopenia grave foi 66,7%.^(^^24^^)^ Smoliner et al.,^(^^25^^)^ no entanto, descreveram, em seu estudo realizado em hospital na Alemanha, com indivíduos idosos internados por situações agudas gerais, utilizando os mesmos critérios adotados nesta investigação, que 25,3% dos pacientes avaliados foram diagnosticados com sarcopenia, entre os quais apenas 18,7% eram severamente sarcopênicos.

O percentual de indivíduos classificados como obesos sarcopênicos (35%) é superior a dados previamente descritos na literatura para pacientes cardiopatas. A maioria dos estudos reportou prevalência abaixo de 10%,^(^^26^^–^^28^^)^ mas a variação na prevalência pode ser bem ampla (0,41%), dependendo da população estudada e da metodologia adotada para definição.^(^^29^^)^ Embora no presente estudo não tenha sido encontrada associação entre OS e parâmetros de risco de doença coronariana, a literatura aponta evidências que sugerem relação. O envelhecimento está associado à redução da atividade física, que, por sua vez, leva a reduções na massa muscular, força muscular e contribui ainda para diminuir o nível de atividade física e a resistência.^(^^3^^,^^30^^,^^31^^)^ A má qualidade muscular, com aumento da infiltração de gordura, pode aumentar ainda mais a inflamação. A diminuição da atividade física pode levar a ganho de peso, aumento da resistência à insulina e aumento da gordura abdominal total com aumentos concomitantes na inflamação. À medida que a massa de gordura aumenta a secreção de leptina e de outras adipocinas e citoquinas, também pode haver contribuição para o desenvolvimento de OS. Este ciclo vicioso entre perda de massa magra e ganho de massa gorda pode levar às condições de sarcopenia e OS.^(^^29^^)^

A sarcopenia foi maior nos homens e nos indivíduos com idade mais avançada, resultados que corroboram dados da literatura, que apontam maiores perdas musculares em homens do que em mulheres. Este dado também foi descrito por Janssen et al.,^(^^10^^)^ que relataram a prevalência de sarcopenia em idosos de 31% para as mulheres e 64% para os homens. Isso acontece porque o homem tem maior perda muscular decorrente do declínio do hormônio do crescimento, fator de crescimento relacionado à insulina (IGF-1) e da testosterona; além disso, eles teriam pior adaptação à perda muscular do que o sexo feminino.^(^^31^^,^^32^^)^ A sarcopenia também aumenta com a progressão da idade, devido a alterações morfofuncionais inerentes ao envelhecimento, como a diminuição da massa livre de gordura e da força muscular, resultando na sarcopenia.^(^^33^^,^^34^^)^

Dos marcadores prognósticos e de risco da doença coronariana, apenas o escore TIMI apresentou associação com a sarcopenia. Portanto, pacientes coronarianos sarcopênicos tiveram maior pontuação do escore, indicando maior risco de complicações quando comparados com os idosos não sarcopênicos. Embora tenhamos verificado este resultado, não foram encontradas, na literatura, análises semelhantes envolvendo esses parâmetros, sendo necessários mais estudos para fundamentar tais achados. De qualquer forma, já foi sugerido que a sarcopenia não é um evento isolado, mas acompanhado por um aumento simultâneo da massa gordurosa.^(^^3^^)^ Esta infiltração de lipídeos desempenha papel na manutenção da sarcopenia, por meio de uma liberação mediada por macrófagos de citocinas pró-inflamatórias e adipocinas, que induzem inflamação crônica. Aqueles com sarcopenia também comumente experimentam deficiência funcional e deficiência física, que provocam redução nos fatores induzidos pela contração muscular com efeito anti-inflamatório − as chamadas “miocinas”. A relativa escassez de miocinas na sarcopenia pode aumentar o risco de DCV. Em geral, a deterioração funcional e a inflamação crônica, bem como a redução de substâncias anti-inflamatórias observadas nas pessoas com sarcopenia, contribuem para o desenvolvimento de resistência à insulina, DM tipo 2, hiperlipidemia e hipertensão, e, eventualmente, aumentam o risco de DCV.^(^^21^^)^ Porém, esse achado ainda deve ser interpretado com cautela, sobretudo porque os demais marcadores não apresentaram associação com a sarcopenia no nosso estudo e também não foram analisados previamente.

Há algumas limitações no estudo que precisam ser apontadas. Por ser um estudo retrospectivo, com análise de prontuários, deve-se considerar a limitação nos registros de alguns dados, bem como ausências de respostas. O tamanho da amostra relativamente pequeno limita o poder estatístico do estudo. Além disso, a amostra procedente de um centro único, com o grupo específico de coronariopatas (pacientes com IAM), restringe a extrapolação dos resultados para outras populações.

## CONCLUSÃO

Ainda são escassos estudos na literatura acerca do problema da sarcopenia e da obesidade sarcopênica com os marcadores de risco de coronariopatias. A elevada prevalência de sarcopenia foi observada nos pacientes coronariopatas, sobretudo em homens e indivíduos mais velhos. A obesidade sarcopênica foi identificada em menor proporção que a sarcopenia, mas ainda acometeu cerca de um terço dos pacientes. Dentre os marcadores de risco e prognóstico, a sarcopenia foi associada apenas ao *thrombolysis in myocardial infarction,* e a obesidade sarcopênica não se associou a nenhum parâmetro. Estudos adicionais são necessários para esclarecer possíveis efeitos da sarcopenia e obesidade sarcopênica como preditores de complicações e prognóstico em pacientes cardiopatas. De qualquer forma, considerando a associação dessas condições com desfechos adversos, fica evidente a importância de sua avaliação rotineira no acompanhamento desses pacientes.
